# Advancing indocyanine green fluorescence flap perfusion assessment via near infrared signal quantification

**DOI:** 10.1016/j.jpra.2024.05.005

**Published:** 2024-05-31

**Authors:** J. Dalli, F. Reilly, J.P. Epperlein, S. Potter, R. Cahill

**Affiliations:** a UCD Centre for Precision Surgery, School of Medicine, UCD, Dublin, Ireland; b Department of Plastic & Reconstructive Surgery, Mater Misericordiae University Hospital, Dublin, Ireland; c IBM Research Europe, Dublin, Ireland; d UCD School of Medicine, UCD, Dublin, Ireland; e Department of Surgery, Mater Misericordiae University Hospital, Dublin, Ireland

**Keywords:** Indocyanine green, ICG, Fluorescence angiography, Free Flaps, Oncoplastic

## Abstract

**Introduction:**

Intraoperative indocyanine green fluorescence angiography (ICGFA) perfusion assessment has been demonstrated to reduce complications in reconstructive surgery. This study sought to advance ICGFA flap perfusion assessment via quantification methodologies.

**Method:**

Patients undergoing pedicled and free flap reconstruction were subjected to intraoperative ICGFA flap perfusion assessment using either an open or endoscopic system. Patient demographics, clinical impact of ICGFA and outcomes were documented. From the ICGFA recordings, fluorescence signal quality, as well as inflow/outflow milestones for the flap and surrounding (control) tissue were computationally quantified *post hoc* and compared on a region of interest (ROI) level. Further software development intended full flap quantification, metric computation and heatmap generation.

**Results:**

Fifteen patients underwent ICGFA assessment at reconstruction (8 head and neck, 6 breast and 1 perineum) including 10 free and 5 pedicled flaps. Visual ICGFA interpretation altered on-table management in 33.3% of cases, with flap edges trimmed in 4 and a re-anastomosis in 1 patient. One patient suffered post-operative flap dehiscence. Laparoscopic camera use proved feasible but recorded a lower quality signal than the open system.

Using established and novel metrics, objective ICGFA signal ROI quantification permitted perfusion comparisons between the flap and surrounding tissue. Full flap assessment feasibility was demonstrated by computing all pixels and subsequent outputs summarisation as heatmaps.

**Conclusion:**

This trial demonstrated the feasibility and potential for ICGFA with operator based and quantitative flap perfusion assessment across several reconstructive applications. Further development and implementation of these computational methods requires technique and device standardisation.

## Introduction

The use of autologous reconstruction via free and pedicled flaps has expanded in recent years. Overall flap survival rates are high (94.2%-97.6%^1^), with reoperation rates reported at 2%-4%^2^ for pedicled flaps (PF) and up to 5% for free flaps.^3^^,^^4^ However, even minor complications can impact patient recovery and delay adjuvant oncological treatment. Close monitoring and early detection of ischaemia improves outcomes, and recent efforts involving indocyanine green fluorescence angiography (ICGFA) intraoperative flap perfusion assessment aim to pre-empt and prevent post-operative complications.^4^, ^5^, ^6^

ICGFA helps visualise flap inflow and outflow via peripherally injected ICG dye and near infrared (NIR) cameras, which highlight ICG as it permeates tissues. Its use is ascendant in plastic surgery in general^7^, ^8^, ^9^ and has been shown to cost effectively reduce complications in breast reconstruction,^10^^,^^11^ with large trials underway.^12^ However, evidence is yet to be accrued in other areas, such as head and neck surgery, where patients typically have multiple co-morbidities^13^ and flap failure may be catastrophic as it can result in exposure of vital structures, vascular anastomotic blowout, pharyngo-oesophageal anastomotic leakage or salivary fistulae.^14^

The uptake and dissemination of ICGFA is, however, hampered by the yet to be established best use of this tool^15^ and interpretation variability has been demonstrated in other surgical specialities.^16^^,^^17^ Additionally, complex optical phenomena dictating NIR signal sensing and presentation, along with inter-system performance heterogeneity, further complicate its clinical exploitation.^18^ There is thus an effort to introduce objective quantification in ICGFA application by digitally measuring the fluorescence intensity as still readings or dynamic curves. Perfusion patterns extracted from the measurements of intensity and timing of fluorescence in relation to inflow and outflow have been demonstrated to be predictive of flap perfusion related complications,^8^ but their application awaits clinical validation.^19^

In this endeavour, we first audited ICGFA use with 2 different NIR camera systems in a tertiary plastic surgery unit with a view to protocolise and clinically introduce them in a series of patients undergoing free and pedicle flaps for head and neck, breast and perineal reconstructions. We subsequently sought to advance ICGFA quantification methodologies to develop objective flap perfusion assessment.

## Methodology

Within an approved clinical trial (Mater Misericordiae University Hospital, Dublin, Ireland 1/378/2092, ClinicalTrials.gov Identifier: NCT04220242), consenting adult patients undergoing pedicled and free flap autologous flap reconstructions during the trial period (May 2021 to May 2022) were sequentially recruited. Patients were excluded if they reported allergies to iodine-containing food/drugs or contrast agents, and if they suffered from severe renal or hepatic impairment.

Following flap raising, and, in the case of free flaps, vascular anastomosis, and prior to skin closure, all ambient lighting was dimmed/blocked, and the NIR camera was positioned to capture the clinically relevant field of view (FOV). Dissolved ICG (0.25 mg/kg Verdye, Diagnostic Green, Germany) was injected intravenously followed by a 10 ml saline bolus while the operators visually interpreted the ICGFA flap perfusion over 4 min with the video display also being recorded for the post hoc analysis. Either an open NIR system (EleVision, Medtronic, Ireland) or a laparoscopic system (Pinpoint, Stryker, USA) with a 30° lens was used for each case. Dynamic software fluorescence boosting capabilities specific to the open system were deactivated via manufacturer directed settings, as these induce baseline fluctuations that preclude *computational* fluorescence quantification. Patient demographics including age, weight, underlying pathology, preoperative (oncological) treatment and outcomes were collected. Post-operative outcomes were detailed from clinical notes and complications were graded according to the Dindo–Clavien classification.^20^

The 4-minute ICGFA recordings from each case were analysed post-operatively using a developed software.^21^^,^^22^ This allowed annotation of regions of interest (ROI) via user drawn boxes on the flap/surrounding tissue, with subsequent simultaneous automatic tracking of these ROIs in the concurrent white light imagery (compensating for movements, such as breathing) and quantification of the changes in fluorescence intensity in greyscale units (g.u.) as a time series (s) from the NIR signal. For all flaps, fluorescence time series were enumerated from ROI of the flap and surrounding/contralateral (control) tissue. From these curves, previously reported^23^ and novel mathematical perfusion parameters were extracted. This included measurement of the period until the flap starts to fluoresce (i.e. pre-perfusion period: *latency* in s) and heterogeneity/homogeneity of fluorescence initiation throughout the flap (the *deviation* from the *median latency: ML*). The maximum brightness (*F_max_* in g.u., a surrogate of ICG concentration) was recorded in g.u. and time for the flap to achieve *F_max_* (from the end of the *latency* period) was identified as *T_max_* (s). The rate of inflow was analysed via the upslope gradient and time to achieve half *F_max_* (*T_1/2_*). The rate of outflow was assessed by the overall *downslope* (g.u./s) gradient and the gradient at 100 s following *F_max_* (*downslope_100_* g.u./s). The previously reported time ratio (*TR,* calculated by dividing *T_1/2_* by *T_max_*^24^*)* was also recorded. To quantitatively compare the quality of the fluorescence reading from each system, the mean signal-to-noise ratio (SNR, window size of 20 readings^25^) was also calculated from the time series for 2 similar cases of delayed oncoplastic breast reconstructions (post-adjuvant therapy and each assessed with separate systems) for ROI from flaps and control tissue (surrounding skin). Means were compared in SPSS (version 27, IBM, USA) via distribution appropriate tests (Mann–Whitney U and Kruskal–Wallis tests with *post hoc* pairwise testing with Bonferroni correction) following normality assessment with Shapiro–Wilk test.

In this study, the software was also advanced to permit selection of irregularly shaped polygons to allow demarcation of the flap contour. Furthermore, within this selected region, the fluorescence signal could be quantified from every pixel (as opposed to a single averaged reading per ROI). *F_max_*, slopes and deviation from *ML* were calculated, and their numerical results were displayed as a heatmap overlay with colours representing values on the original white light image as a summarising full flap augmented representation of the ICGFA. Slope assessments were improved to compensate for image saturation by measuring the inflow gradient at 2 to 4 s and outflow gradient at 90 to 100 s post-latency.

## Results

Intraoperative ICGFA was carried out on all recruited patients (n = 15; 6 males and 9 females) and no ICG-related complications were recorded. The cohort featured patients aged 44-90 years (mean 64.6 years), who received a mean ICG dose of 17.5 mg (mean patient weight = 69.9 kg). Most flaps were for oncological reconstruction, among which 53% (n = 8) were for head and neck cancers and 40% (n = 6) for breast cancers. One patient underwent an inferior gluteal artery myocutaneous flap (IGAM) reconstruction for perineal Crohn's disease. Among the 15 cases, 6 (40%) had previously received radiotherapy and 3 (20%) had also received systemic chemotherapy as part of their treatment. Most patients underwent free flaps (FF) (n = 11, 73%; Table 1).

**Table 1:** Patient demographics, changes in clinical management following ICG perfusion angiography and complications graded using the Dindo–Clavien classification.^20^

Patient demographics and data	Overall	Head and neck	Breast	Perineal
N	15	8	6	1
M: F	06:09	06:02	06:00	00:01
Mean age (years)	64.6	74.4	55	44
Mean weight (kg)	69.9	74	67.7	50
Malignant versus benign	14:1	8:0	6:00	00:01
Open: laparoscopic	12:3	6:2	5:1	1:00
Free: pedicled flap	10:5	7:1	3:3	0:1
Change in management % (n)	33.3% (5)	37.5% (3)	33% (2)	0
Edges trimmed	(4)	(2)	(2)	0
Failed inflow	(1)	(1)	0	0
Post-op complications (Dindo–Clavien grade)	I (1) III (3) V (1)	III (1) V (1)	I (1) II (2) III (1)	none
Seroma (I)	(1)	0	(1)	0
Intervention under general anaesthesia (dehiscence and haematoma evacuation) (IIIb)	(3)	(1)	(2)	0
Distant metastasis (V)	(1)	(1)	0	0

Regarding angiogram acquisition, overall, a suboptimal ‘flooded’ angiogram was noted when the operative field was covered in blood during the injection phase for an upper airway reconstruction, precluding visual and quantitative interpretation (n = 1). The laparoscopic system was noted to be more susceptible to ambient lighting artefacts (e.g. surgical microscope; n = 1) causing artefactual fluorescence on the flap and also resulted in a weak signal when the camera was far enough to visualise the whole flap, necessitating piecemeal flap visualisation closer to the tissue. Simultaneously, the open system demonstrated signal saturation (i.e. intensity exceeding the detectable threshold of the system, n = 6). This resulted in a signal plateau, limiting quantitative assessment on ROI and whole flap level.

**Figure 1:**
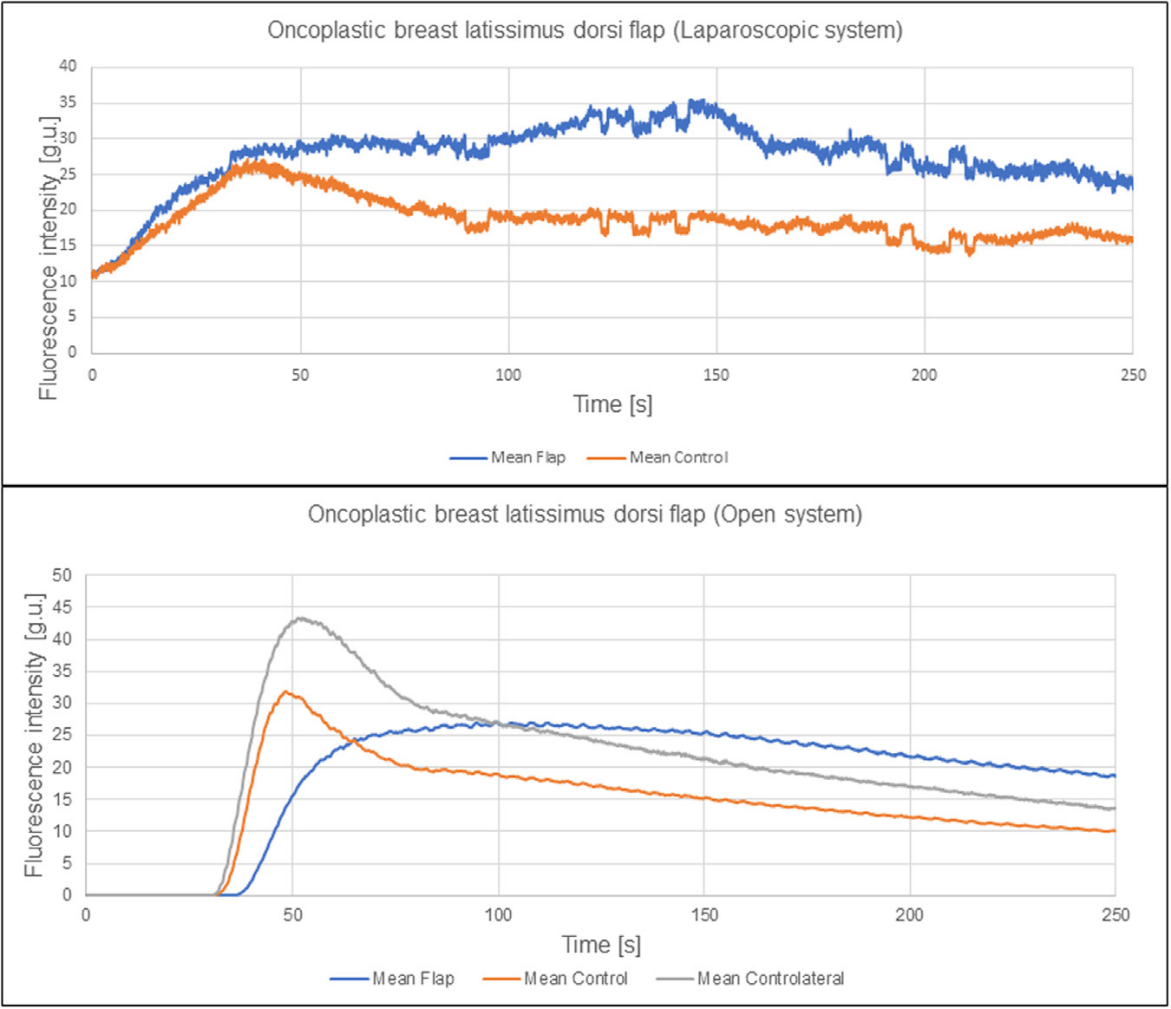
Curves for time (in seconds, s) versus mean fluorescence intensity curve (in greyscale units, g.u.) for the flap, control and contralateral (Medtronic only) for 2 oncoplastic breast latissimus dorsi reconstructions using the laparoscopic and dedicated open systems. Signal-to-noise ratio was used to quantify visual discrepancy.

**Signal-to-noise ratio (SNR):** The plots quantified from the recordings with the open system subjectively appeared smoother than those from the laparoscopic camera (Figure 1). Objectively, SNR was greater (better) for the dedicated open system than the laparoscopic system (flap 212.1 ± 103.2 vs 72.6 ± 26.8 and control 188.2 ± 79.9 vs 59.2 ± 19 p < 0.001 for both comparisons).

**Figure 2:**
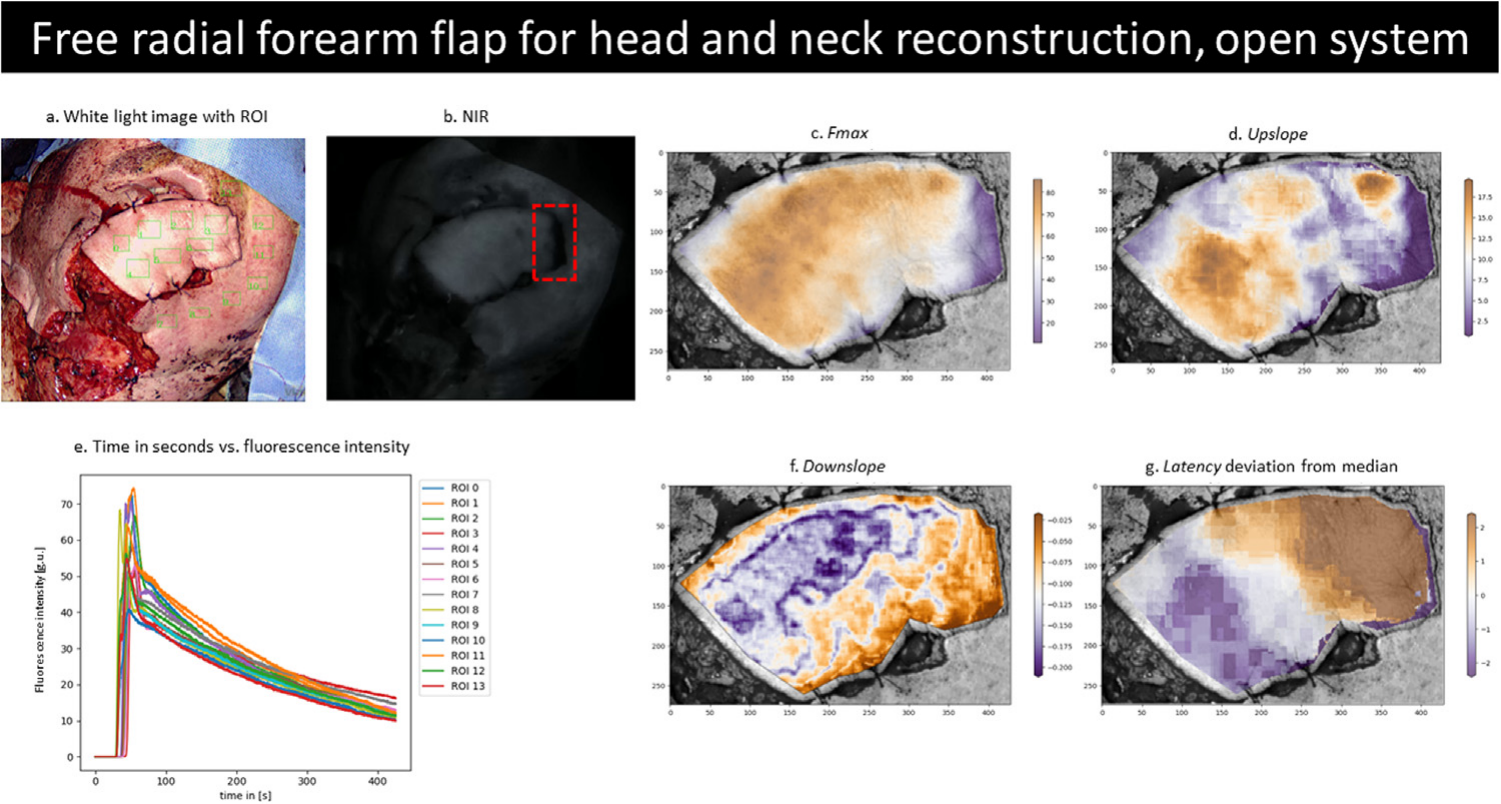
Composite image showing a free radial forearm flap deployed to reconstruct a head and neck defect (a) in white light with sampled regions of interest (ROI, green boxes), (b) NIR view with an area (red box) of lower fluorescence uptake, which was trimmed, and a plot (e) of the time (in seconds, s) versus fluorescence intensity curve (in greyscale units, g.u.). Full-screen FOV with per-pixel curve analysis for *F_max_* (c), *upslope* (d) *downslope* gradients (f) and *latency deviation* from *median* (g). Legends to the right of the heatmaps (c, d, f and g) illustrate the equivalent value for the colours, ascending from purple to orange.

Head and neck reconstructions: This cohort (male: female 6:2) featured patients with a mean age of 74.4 years undergoing oncoplastic reconstruction. Seven cases were reconstructed with FF, and 1 was a local extended cervicofacial flap. Five patients had ICGFA using the open NIR camera system and 2 using the laparoscopic camera. Visual ICGFA interpretation impacted intraoperative management in 3 (38%) cases. In 2 cases, while the flap generally fluoresced, the edges of the flap did not; thus, these parts were trimmed (Figure 2). In 1 case, the entire flap appeared malperfused by ICGFA and anastomotic arterial inflow insufficiency was confirmed using Doppler examination, and thus the arterial anastomosis was redone. One patient suffered donor site dehiscence post-operatively following harvest of an extended anterolateral thigh free flap and required re-grafting.

ROI-based fluorescence quantification of a radial forearm FF for reconstruction of a total lip defect following resection of a recurrent leiomyosarcoma showed that inflow started later in the flap versus the surrounding tissue (*latency* 39.91 ± 3.04 vs 32.50 ± 3.88 s, p = 0.014; Table 2 and Figure 2). Besides the overall later start, flap inflow initiation was also more heterogenous (greater deviation from ML, p = 0.014) compared to the surrounding tissue. The flap shone brighter (*F_max_* 66.18 ± 8.20 vs 55.34 ± 8.19 g.u., p = 0.039), outflow (gradient) was brisker overall and 100 s after the peak, (p = 0.039 for both) and the TR ratio was reduced for the flap (0.20 ± 0.03 vs 0.25 ± 0.05, p = 0.028).

**Table 2:** Curve fluorescence criteria derived from ROI-based assessment for a head and neck reconstruction using a free radial forearm flap and a perineal reconstruction using an IGAM flap.

	Free radial forearm flap (head and neck)			IGAM flap (perineal)		
Fluorescence criteria	Open NIR platform			Open NIR platform		
	Mean ± SD		p Value mwu	Mean ± SD		p Value mwu
	Control	Flap		Control	Flap	
ROI (n)	9	6		3	6	
*Latency* (s)	32.50 ± 3.88	39.91 ± 3.04	0.014*	38.42 ± 2.90	25.32 ± 1.15	0.020*
*Median latency* (ML in s)	34.59		n/a	36.91		n/a
*ML* deviation (s)	−2.09 ± 3.88	5.32 ± 3.04	0.014*	1.52 ± 2.90	−11.59 ± 1.15	0.020*
*F_max_* (g.u.)	55.34 ± 8.19	66.18 ± 8.20	0.039*	27.13 ± 2.91	33.92 ± 9.31	0.121
*T_max_* (s)	13.17 ± 3.20	13.92 ± 0.27	0.697	22.76 ± 10.23	10.34 ± 1.76	0.020*
*Upslope* (g.u./s)	4.44 ± 2.95	4.39 ± 0.57	0.093	1.12 ± 0.50	2.75 ± 0.69	0.020*
*T_1/2_* (s)	3.22 ± 0.69	2.79 ± 0.46	0.121	5.15 ± 1.70	3.53 ± 0.58	0.039*
*Downslope* (g.u./s)	0.11 ± 0.02	0.14 ± 0.03	0.039*	0.09 ± 0.01	0.11 ± 0.03	0.197
*Downslope_100_ (*g.u./s)	0.24 ± 0.07	0.34 ± 0.06	0.039*	0.09 ± 0.02	0.21 ± 0.07	0.071
*TR*	0.25 ± 0.05	0.20 ± 0.03	0.028*	0.25 ± 0.12	0.34 ± 0.02	0.439

p Values for the statistical comparisons were obtained via Mann–Whitney U Test (mwu).

⁎ Denotes statistically significant differences (p < 0.05)

**Oncoplastic breast reconstructions:** These female patients (n = 6, mean age 55 years) underwent oncoplastic breast reconstructions with equal proportions undergoing free deep inferior epigastric flap (DIEP) and pedicled latissimus dorsi (LD) flap reconstructions. The laparoscopic system was used once. Within these cases, ICGFA altered management twice, guiding trimming of edges which did not fluoresce. Two patients required return to theatre and re-intervention under general anaesthesia (Grade III). One patient required evacuation of a haematoma, while the other patient suffered DIEP dehiscence in the setting of previous radiotherapy. This dehiscence required debridement of necrotic edges and seroma drainage under general anaesthesia. A donor site seroma was recorded for an LD reconstruction.

**Figure 3:**
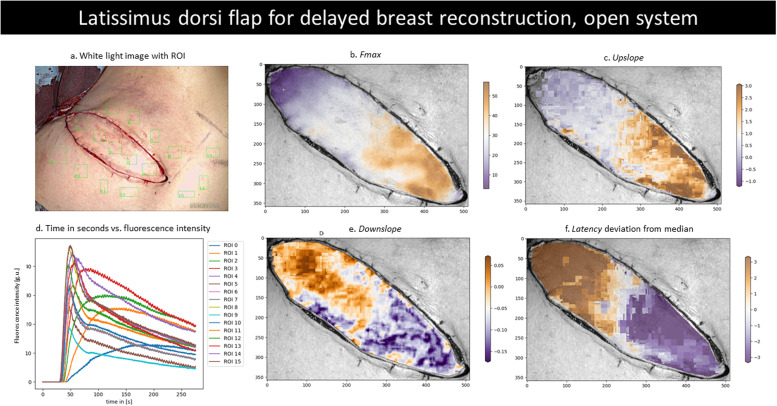
Composite image showing a latissimus dorsi flap deployed as delayed oncoplastic reconstruction following breast surgery in white light (a) with sampled regions of interest (ROI) and a plot (d) of the time (in seconds, s) versus fluorescence intensity (in greyscale units, g.u.). Full-screen field of view with per-pixel curve analysis for *F_max_* (b), *upslope* (c) *downslope* (e) gradients and *latency deviation* from *median* (f). Legends to the right of the images (b, c, e and f) illustrate the equivalent value for the colours, ascending from purple to orange.

ROI-based quantification of a delayed breast reconstruction with an LD flap following chemo-radio therapy for breast cancer using the open system showed that the ipsilateral native skin was not significantly different from the contralateral side (despite previous radiotherapy; Table 3 and Figure 3). When comparing the flap (F) to the control (C) and contralateral (CL) sides, the flap started to fluoresce later (*latency* F:49 ± 13.9 vs C:34.2 ± 0.9 and CL: 36.4 ± 2.2 s, p = 0.006 and on *post hoc* p = 0.042 and 0.008). Also, the onset of fluorescence was less uniform than the surrounding skin and contralateral breast with a greater deviation from *ML* (F:12.2 ± 13.8 vs C: −0.4 ± 2.2 and CL: −2.4 ± 0.9 s, post hoc p = 0.042 and 0.008; Table 3). Once the inflow started, fluorescence intensity climbed more slowly than the surrounding tissue (F vs C *upslope* p = 0.013 and *T_1/2_* p = 0.001), resulting in the attainment of a peak intensity later (T*_max_* p = 0.002), but with a peak brightness which was not different from its surroundings.

**Table 3:** Curve fluorescence criteria derived from ROI-based assessment for 2 ICGFA following 2 latissimus dorsi oncoplastic breast reconstruction and the p values for the statistical comparisons were obtained via Kruskal–Wallis (kw), *post hoc* testing and Mann–Whitney U Test (mwu).

Latissimus Dorsi flaps for breast Oncoplastic Reconstruction										
Fluorescence criteria	Open NIR Platform							Laparoscopic system and 30° scope		
	Mean ± SD			p Value kw	Post hoc testing			Mean ± SD		p Value mwu
	Control	Flap	Contralateral		Contralateral vs control	Contralateral vs flap	Control vs flap	Control	Flap	
ROI(n)	8	5	3					4	3	
*Latency* (s)	36.39 ± 2.16	49.02 ± 13.78	34.18 ± 0.92	0.006*	0.718	0.008*	0.042*	13.76 ± 3.86	13.27 ± 5.18	1.000
*Median latency* (ML in s)	36.82			n/a	n/a			15.95		n/a
*ML deviation* (s)	−0.43 ± 2.16	12.20 ± 13.78	−2.64 ± 0.92	0.006*	0.718	0.008*	0.042*	−2.19 ± 3.86	−2.68 ± 5.18	1.000
*F_max_* (g.u.)	32.65 ± 8.43	30.27 ± 11.81	44.26 ± 2.96	0.079	n/a			28.89 ± 2.88	35.84 ± 0.71	0.034*
*T_max_* (s)	12.25 ± 1.28	70.04 ± 38.24	19.39 ± 3.52	0.002*	0.211	0.956	0.002*	26.47 ± 5.87	132.67 ± 4.79	0.034*
*Upslope* (g.u./s)	2.23 ± 0.53	0.64 ± 0.69	2.08 ± 0.50	0.014*	1.000	0.173	0.013*	0.51 ± 0.15	0.15 ± 0.00	0.034*
*T_1/2_* (s)	4.31 ± 0.58	15.44 ± 9.85	5.82 ± 0.00	0.002*	0.263	0.749	0.001*	11.68 ± 1.42	16.73 ± 5.88	0.157
*Downslope* (g.u./s)	0.10 ± 0.03	0.08 ± 0.04	0.15 ± 0.01	0.041*	0.162	0.036*	1.000	0.12 ± 0.01	0.19 ± 0.01	0.034*
*Downslope_100_* (g.u./s)	0.17 ± 0.04	0.09 ± 0.06	0.23 ± 0.02	0.011*	0.318	0.009*	0.177	0.10 ± 0.02	0.11 ± 0.02	0.480
*TR*	0.35 ± 0.03	0.22 ± 0.05	0.31 ± 0.05	0.009*	0.867	0.468	0.006*	0.45 ± 0.07	0.13 ± 0.05	0.034*

⁎ Denotes statistically significant differences (p < 0.05, with Bonferroni correction for *post hoc* testing).

Heatmaps presenting the above-described metrics were generated, permitting the presentation of the dynamic data in summarising still images (Figures 2 and 3).

## Discussion

Currently, flap perfusion assessment is based on subjective clinical acumen and Doppler ultrasound, with ICGFA potentially offering an additional decision-making tool, being especially useful where post-operative clinical monitoring of the flap is difficult. Selective ICGFA used only in grey cases would seem more efficient, but acquiring such visual ICGFA interpretative competence requires experiential learning, incrementally accrued by routine use.^26^ ICGFA quantification may support visual interpretation and over time could permit the development of thresholds or artificial intelligence (AI) to predict flap compromise at the instance of the index operation. Such tools could obviate ICGFA learning curves and reduce the need for unplanned flap salvage procedures.

This case series demonstrated the safe and feasible clinical deployment of ICGFA across several anatomical regions and reconstructive applications and shows that quantification signals can be extracted on an ROI and full-screen basis. However, it also identified issues in current practice, especially regarding objective signal quantification. System selection is an early decision that a clinical or research institution must take, as this impacts visual and computational interpretation, as well as the clinical protocol. The now common inclusion of NIR features in readily available laparoscopic systems might encourage their opportunistic use by plastic surgeons as an alternative to the capital investment of a dedicated open system. Indeed, the use of laparoscopic NIR cameras for extracorporeal bowel perfusion assessment is prevalent in clinical practice and the literature.^27^

Although we show this is feasible, laparoscopic systems demonstrate an overall weaker signal and in particular at the peripheries of the image.^18^ Angled scope configurations were used as they are easier to handle compared to the 0° alternatives (as these need to be held perpendicular to the target tissue), but have a narrower FOV.^18^ This limited FOV precludes assessment of normal surrounding skin as control tissue (necessary for quantitative methodologies as ICG pharmacokinetics are dependent on patient physiology,^28^^,^^29^ drugs^30^ and anaesthesia^31^). We have also shown that the sensed fluorescence signal is buried in background fluorescence/noise. This lower SNR results in a curve which is less smooth, complicating the accurate identification and measurement of the relevant perfusion parameters (e.g. *F_max_*).

In contrast, the open system demonstrated here offered consistent distance-intensity performance and a flexible trolley-mounted arm (obviating the needs for sterile handling)^18^ providing a stable image but was prone to image saturation. This highlights the importance of dosing, which results in greater fluorescence intensity with ICG concentration up to 1.6 mg/kg.^32^ For this system, our findings support the working guidance of the International Society for Fluorescence Guided Surgery (ISFGS) which recommends a weight independent dose of 3 ml for mastectomy flap perfusion assessment^15^ (or a dose of 0.11 mg/kg for a *reference* male^33^ of 70 kg). Although IFSGS admit that further research on dosing is needed and is yet to specify a dose for FF reconstruction, it is likely that a similarly reduced dose would be suitable for this system (but is likely too low for the laparoscopic one). The only case suffering a complication attributable to poor flap perfusion with the open system demonstrated an over-saturated angiogram which might have hampered the visual interpretation of the angiogram (potentially masking subtle changes in perfusion at the periphery); this further supports the need for system-specific protocols.

Regarding quantification, although the open system used here provides ‘smart’ adaptation of the signal gain (dynamically correcting for saturation), this allows relative ‘spot’ fluorescence quantification (compared to other areas on screen) from still ICGFA images. However, a study using inbuilt spot fluorescence sampling on other open systems (SPY-QP, Stryker, USA) for prediction in implant based oncoplastic breast reconstruction only resulted in modest sensitivities, specificities and negative predictive values.^34^^,^^35^ This could be due to errors in timing, as it is difficult to standardise the inflow/outflow instance without full curve sampling, or because of arbitrary reference point selection for relative comparisons.^19^

Our methods of dynamic full curve assessment seek to overcome timing errors and full flap assessment portends to improve on arbitrary user guided regional sampling. Furthermore, metrics based on chronology should not be susceptible to variations in fluorescence intensity due to camera positioning.^36^ Although the camera may indeed be fixed, in this study, the working distance was not fixed and was dictated by the ability to visualise the flap and surrounding control tissue within the FOV. This was supported by evidence showing that within the limits of normal use, camera angulation does not impact NIR performance^18^ and that standardisation protocols including set distances may impact user interpretation consistency.^37^ The selected open system also demonstrates consistent NIR performance across a range of distances.^18^

This study also presents novel perfusion metrics. Previously reported work associated absolute *latency* with colorectal complications from the instance of ICG injection.^38^^,^^39^ However, rather than focusing on the absolute *latency* (which is difficult to standardise as it requires synchronising ICG injection and manual ICGFA recording) this work presents a comparison between the flap and control *latencies* and crucially the *deviation* from the ML*,* which is independent of the timing of injection. Pixel-level quantification also permits this novel metric to present inflow heterogeneity. Another novel aspect of this work is the assessment of inflow and outflow gradients in relation to the ‘take off’ at the end of the latency as opposed to the peak, which is not discernible in saturated angiograms as it is replaced by a plateau.

Overall, it would be impossible to visually appreciate complex mathematic metrics such as *TR (T_1/2_: T_max_*), which has demonstrated complication predictive associations in gastrointestinal surgery,^24^ without novel tools to quantify and present this expansive data. This variable demonstrated significance in our data when comparing the pedicled flap with control tissue, and the heatmap capabilities presented here allow such presentation of simple and compound metrics, summarising the whole inflow-outflow curve across the whole flap into a still image. Although these are quantitative representations, users are more likely to interact with colours than with numbers in a 2-dimensional matrix; hence, the impact of these images on inter-user variability requires separate assessment.

This work thus demonstrates the feasibility of assessing these established and novel quantitative ICGFA metrics, as well as advances in the presentation of these complex data across several flap types. However, this same flap heterogeneity, and the single reported perfusion related complication precludes meaningful clinically translatable statistical associations, overall thresholds or intra-patient flap versus control ratios. These computational assessments have also been carried out retrospectively, although from a technical perspective, there is no restriction to run these in theatre on a laptop or potentially directly on the camera system's computer. Thus, the general future application of the demonstrated methodologies is to be clinically deployed alongside visual assessment in outcomes trialling ICG use for specific flap types, similar to the upcoming FAFI trial,^12^ for quantitative comparisons or training of AI.

Indeed, video based ICGFA visual and quantitative assessment does offer opportunities for inflow-outflow assessment, complex maths and metadata presentation, but it also necessitates video recordings of sufficient quality and better-quality imagery could have changed our findings (especially regarding saturation). Bleeding was also noted to flood the field with fluorescence, and thus where this is predicted, suction tubing should be placed pre-emptively. Other potential issues include video interruption due to instrument and hand intrusion. In reality, continuous assessment beyond 3 1/2 min (half-life of ICG),^40^ is needed for adequate outflow assessment, which is important as venous stasis and thrombosis is attributed as the leading cause of FF failure.^41^ These issues, however, can be overcome through practice and technique adaptation, including assimilation of the learnings reported here regarding system selection (e.g. regarding FOV), dosing and videography.

## Conclusion

Overall, this work shows that the clinical deployment of ICGFA is feasible, safe and potentially alters clinical outcomes across different reconstructive settings. Furthermore, signal quantification is feasible when included at the whole screen level and offers promise of objective computational perfusion assessment. However, practice, equipment and technique adaption and standardisation are required for investigative and clinical uptake of ICGFA in reconstructive surgery.

## Conflicts of Interest

Ronan A Cahill is named on a patent filed in relation to processes for visual determination of tissue biology, receives speaker fees from Stryker Corp, Ethicon/J&J and Olympus, research funding from Intuitive Corp, consultancy fees from Arthrex, Diagnostic Green, Distalmotion and Medtronic (Touch Surgery) and holds research funding from the Irish Government (DTIF) in collaboration with IBM Research in Ireland, from EU Horizon 2020 in collaboration with Palliare and Steripak, from Horizon Europe in collaboration with Arctur, and from Intuitive and Medtronic for specific research and development awards.

Dr J Dalli was employed as a researcher in the DTIF, is named on a patent filed by University College Dublin concerning technologies related to the subject matter of this study and is a recipient of the TESS scholarship.

At the time of writing Dr J P Epperlein was a full-time employee of IBM Research, a division of IBM Corporation. IBM Corporation provides technical products and services world-wide to government, healthcare and life- sciences companies. He is named on filed and granted patents concerning technologies related to the subject matter of this study.

Drs F Reilly and S Potter report no disclosures.
